# Cellular Metabolic Heterogeneity and Therapeutic Targeting in Rheumatoid Arthritis

**DOI:** 10.3390/cells15211949

**Published:** 2026-10-26

**Authors:** Sheng Chen, Yufan Luo, Qiuzhi Zhou, Hong Chen

**Affiliations:** 1 Department of Rehabilitation, Tongji Hospital, Tongji Medical College, Huazhong University of Science and Technology, Wuhan 430030, China; cs2cc0602@petalmail.com (S.C.); luoyufan1027@gmail.com (Y.L.); 2 Stem Cell Research Center, Tongji Hospital, Tongji Medical College, Huazhong University of Science and Technology, Wuhan 430030, China; 3 Hubei Key Laboratory of Neural Injury and Functional Reconstruction, Huazhong University of Science and Technology, Wuhan 430030, China

**Keywords:** rheumatoid arthritis, immunometabolism, synovial fibroblasts, metabolic heterogeneity, metabolic reprogramming

## Abstract

**Highlights:**

**What are the main findings?**

- Metabolic defects in rheumatoid arthritis differ across cell populations and tissue environments.
- PFKFB3 inhibition may have opposing effects in synovial fibroblasts and T cells.

**What are the implications of the main findings?**

- Metabolic targeting should account for metabolite transport and tissue location while preserving protective immune functions.
- Durable metabolic reprogramming requires persistent functional change after drug withdrawal.

**Abstract:**

Metabolic abnormalities in rheumatoid arthritis (RA) differ across cell populations and anatomical compartments. Glycolytic synovial fibroblasts coexist with blood T-cell populations showing impaired glycolytic induction or mitochondrial function, while macrophage metabolism can sustain inflammation or tissue protection. These differences complicate systemic metabolic inhibition. This narrative review integrates human RA studies, experimental arthritis and mechanistic work in other systems to identify dependencies that support defined pathogenic functions. We examine metabolite transport, nutrient adaptation and nutrient and energy sensing, distinguishing causal perturbation from descriptive signatures and preclinical hypotheses from clinical efficacy signals. Systemic cardiometabolic disease and nutritional interventions provide a clinical context for interpreting local metabolic findings. Small trials of repurposed drugs suggest possible benefit during treatment, but do not establish durable metabolic correction. We propose that a claim of reprogramming requires persistent functional change after drug removal, exclusion of residual exposure and selective cell loss, and preservation of immune regulation and host defence. These criteria could guide more selective metabolic interventions and their evaluation in patient tissue.

## 1. Introduction

Rheumatoid arthritis (RA) is sustained by interactions among adaptive immunity, innate inflammatory cells and tissue-resident stromal populations. Responses to therapies targeting cytokines, lymphocyte costimulation and B cells have established the importance of these mechanisms, although persistent synovitis and relapse remain clinical problems [1,2]. These cellular responses also depend on metabolism: cytokine production, migration, matrix remodelling and immune regulation require coordinated control of substrate use and organelle function. Recent reviews have examined metabolic checkpoints in RA pathogenesis [3]. The unresolved issue is which alterations enable pathogenic functions, which compensate for environmental stress, and which can be modified without disabling protective immunity.

Human synovial atlases have revealed substantial variation in cellular composition and activation states across patients. These observations challenge models that treat the inflamed joint as a single, uniformly glycolytic compartment. At the same time, studies of patient-derived T cells demonstrate that inflammatory potential can arise from inadequate glycolytic induction or mitochondrial metabolite production, rather than from generalized metabolic acceleration. The same metabolic intervention may therefore have different effects across cell types and across activation states within a lineage [4,5,6].

We examine metabolic abnormalities in the synovial environment, their functional consequences in individual cell populations and their implications for treatment. The central question is whether a metabolic intervention can suppress a defined pathogenic function while preserving protective responses in the same tissue. We also consider what would justify a claim of durable reprogramming. Here, this term refers to a functional change that persists after drug removal, with preserved viability and appropriate immune competence. It is a hypothesis to be tested, rather than an established explanation for RA remission.

### 1.1. Review Approach and Evidence Selection

This is a focused narrative synthesis, not a systematic review or meta-analysis. For the revision, targeted web-assisted searches of PubMed-indexed records and journal websites were completed on 20 October 2026, without a lower publication-year restriction. Topic queries combined rheumatoid arthritis with metabolism, glycolysis, mitochondria, regulatory T cells, succinate, itaconate, amino acid metabolism, cardiometabolic multimorbidity, ketogenic diet and spatial metabolomics.

### 1.2. Terminology and Functional Interpretation

A metabolic signature is an observed pattern of metabolites, proteins or transcripts and need not indicate altered flux. A metabolic defect is an experimentally demonstrated impairment relative to an appropriate comparator. A dependency is a requirement for a specified function established by perturbation, preferably with rescue. An adaptation is a response that sustains survival or function under an environmental constraint; it can support pathogenic or beneficial activity. We use protective function for a measured outcome such as efferocytosis, barrier maintenance or regulatory suppression. A remission-associated population is described as associated with remission unless protection has been demonstrated functionally. These categories are not interchangeable.

## 2. The Synovial Metabolic Environment

### 2.1. Hypoxia and Spatial Organization

Direct measurements in inflammatory arthritis link low synovial oxygen tension with inflammatory activity, supporting hypoxia as a biologically relevant feature of the diseased joint. These measurements capture local oxygen tension at the time of sampling, leaving its spatial and temporal variation incompletely defined. Vascular density alone is also a poor measure of oxygen delivery. Perfusion, diffusion distance and local consumption jointly determine cellular oxygen availability [7].

Hypoxia can also damage the machinery needed for subsequent metabolic adaptation. In inflammatory arthritis tissue, lower oxygen tension was associated with a greater burden of mitochondrial deoxyribonucleic acid (DNA) mutations and oxidative stress; hypoxic exposure induced related abnormalities in cultured synoviocytes [8]. These findings link hypoxic exposure to organelle injury, whereas some circulating RA T cells exhibit low reactive oxygen species. Oxidative injury in synovial tissue can therefore coexist with reductive stress in circulating lymphocytes.

Single-cell studies first resolved previously unrecognized synovial fibroblast populations and subsequently mapped inflammatory mediators to discrete stromal, myeloid and lymphoid states [9,10]. The 2023 multimodal RA atlas extended this framework to heterogeneous inflammatory tissue subtypes, while a 2025 spatial study identified a lymphatic vessel endothelial hyaluronan receptor 1 (LYVE1)-positive macrophage network associated with therapeutic response [4,11]. Early treatment-naive cohorts independently linked synovial cellular and molecular signatures to disease activity, response and structural progression [12,13]. Changes in cell composition can alter a metabolic measurement from an unsorted biopsy. The atlases help assign such changes to candidate populations, while oxygen distribution and metabolic flux require separate measurements.

Spatial position can instruct cellular identity as well as reflect it. Endothelial-derived Notch signals establish gradients of NOTCH3 activity in perivascular and sublining fibroblasts; genetic or antibody-mediated interference reduced arthritis pathology in experimental systems [14]. Notch signalling thus links vascular position to stromal function. Whether these spatially defined states also differ in metabolic dependence remains to be established through functional measurements.

To test these relationships prospectively, local oxygen tension could be measured with a calibrated tissue probe and co-registered with biopsy position, vascular imaging and histology [7]. Hypoxia-responsive transcription or staining would provide complementary evidence of exposure, rather than a substitute for oxygen measurement. Extracellular pH should be measured promptly with calibrated microelectrodes or extracellular ratiometric probes under controlled temperature and carbon dioxide. Oxygen exposure, sampling delay and tissue handling should be recorded because ex vivo measurements may differ from the intact joint.

### 2.2. Nutrient Availability and Metabolite Exchange

Nutrient limitation is mechanistically relevant, but competition among cell populations remains incompletely quantified. RA macrophages can resist glucose withdrawal through a programme involving C-C motif chemokine ligand 18 (CCL18), regulatory factor X5 (RFX5) and glutamate dehydrogenase 1 (GLUD1) that supports glutamate utilization [15]. This identifies a survival response to glucose shortage, but the source and magnitude of that shortage in intact tissue remain uncertain. Demonstrating competition would require showing that consumption by one population limits substrate availability and changes the function of another. Enzyme or transporter expression alone cannot identify the source of metabolites measured in synovial fluid.

Lactate provides a more experimentally developed example of intercellular metabolic signalling. In an RA-relevant study, solute carrier family 5 member 12 (SLC5A12)-dependent uptake altered cluster of differentiation 4 (CD4) T-cell function and tissue retention, linking extracellular lactate to adaptive immune behaviour. Lactate transport and extracellular acidification have separable effects. Sodium-coupled SLC5 transporters and proton-linked monocarboxylate transporters have different transport properties, and extracellular pH has effects that should be experimentally separated from those of lactate itself [16].

Intercellular exchange is best tested by combining metabolite measurements with selective perturbation of the producer or recipient. Spatial localization and isotope tracing can then distinguish substrate transfer from independent metabolic changes in neighbouring cells.

A prospective competition experiment would pair matched blood, synovial fluid and tissue metabolite measurements with cell-resolved uptake or isotope labelling. In an explant or coculture, selective inhibition of one population should increase substrate availability and alter a neighbouring population’s function; substrate add-back would test whether deprivation explains the effect. Spatial proximity alone cannot establish competition. Glucose or glutamine depletion, lactate release and pH should therefore be measured alongside cell number and viability [17,18].

### 2.3. Systemic Metabolism and Cardiometabolic Multimorbidity

Local metabolic findings also sit within systemic disease. In a cross-sectional study of 757 patients, Ruscitti et al. defined cardiometabolic multimorbidity as at least two of hypertension, dyslipidaemia and type 2 diabetes. This subgroup had less frequent remission and more previous biologic treatment failure [19]. The association identifies clinical heterogeneity; it does not establish that a specific synovial metabolic pathway caused either comorbidity or treatment resistance.

A translational implication is to record adiposity, glycemic and lipid status, cardiovascular disease, renal function and medication exposure when interpreting circulating metabolites or selecting patients for metabolic trials. Paired systemic and tissue measurements could test whether a biomarker tracks joint biology, comorbidity or both. Clinical outcomes should include cardiometabolic effects as well as arthritis activity, without assuming that improvement in one compartment proves correction in the other.

## 3. Metabolic Requirements of Synovial and Circulating Cells

### 3.1. Macrophage Metabolism in Inflammation and Resolution

The glycolytic M1 and oxidative M2 phenotypes describe selected culture conditions and capture only part of RA macrophage diversity. Fate-mapping and imaging identified locally renewing C-X3-C motif chemokine receptor 1 (CX3CR1)-positive lining macrophages that form a joint barrier in experimental arthritis [20]. Human tissue studies identified MER tyrosine kinase (MerTK)-positive states associated with resolution and lower flare risk during remission [21]. The mouse barrier experiments and human remission associations provide different evidence of protection. Neither establishes a uniform fuel requirement for these populations.

Metabolic abnormalities are also detectable in circulating cluster of differentiation 14 (CD14)-positive monocytes from patients with RA, including in individuals studied before clinical disease manifestation. RA monocytes showed enhanced mitochondrial respiration alongside increased glycolytic machinery and glucose-dependent inflammatory responses. Concurrent increases in respiration and glycolysis argue against a uniform switch between these pathways. The findings before clinical onset also suggest that myeloid abnormalities can precede exposure to an inflamed joint. The contribution of circulating monocytes to specific synovial macrophage states nevertheless remains unresolved [22].

Macrophage studies further distinguish the catalytic and signalling functions of metabolic enzymes. Integrated tracing and transcriptional analyses identified stimulus-dependent tricarboxylic acid (TCA) cycle remodelling and compensatory reactions in polarized murine macrophages [23]. Succinate can regulate interleukin-1β (IL-1β) through hypoxia-inducible factor-1α (HIF-1α), while its mitochondrial oxidation can promote inflammatory reactive oxygen species [24,25]. Pyruvate kinase M2 (PKM2) likewise has separable catalytic and signalling roles: enforcing its active conformation reduced HIF-1α-associated inflammatory output in lipopolysaccharide (LPS) models, whereas reducing PKM2 expression attenuated macrophage activation and pristane-induced arthritis [26,27]. Their effects are compatible with distinct catalytic and signalling roles of PKM2; enzyme activation and depletion need not produce opposite inflammatory outcomes.

Amino acid catabolism provides additional support for pathogenic macrophage functions. Branched-chain amino acid aminotransferase 1 (BCAT1) inhibition reduced respiratory and glycolytic activity and altered the aconitate decarboxylase 1 (ACOD1, also called immune-responsive gene 1 or IRG1)–itaconate programme in human macrophages. Inhibition also reduced inflammation in experimental arthritis [28]. In RA synovial macrophages, CCL18-induced RFX5 coordinated GLUD1-dependent survival during glucose withdrawal with increased human leukocyte antigen DR (HLA-DR) expression and antigen presentation [15]. This adaptation may allow macrophages to survive nutrient stress while continuing to stimulate lymphocytes. Antigen presentation and survival are therefore relevant outcomes alongside cytokine production.

Macrophages with repair-associated markers can lose protective function. A 2026 study showed that patient-derived selenoprotein P (SELENOP)-high, MerTK-positive, cluster of differentiation 206 (CD206)-positive macrophages could undergo complement component 1q (C1q)-associated metabolic exhaustion. Mitochondrial sterile alpha and Toll/interleukin-1 receptor motif-containing 1 (SARM1) activity depleted nicotinamide adenine dinucleotide (NAD) and promoted PANoptotic inflammatory cell death, integrating pyroptotic, apoptotic and necroptotic machinery; SARM1 inhibition reduced synovitis in the experimental system [29]. Surface-marker expression alone would miss this loss of function. Conversely, interleukin 6 (IL-6) inhibition can influence Axl and MerTK biology, suggesting that cytokine therapy can also affect pathways involved in resolution [30]. Macrophage phenotyping is consequently more informative when paired with measurements of organelle integrity and repair function.

Inflammatory history may constrain reversibility. In polarization models, mitochondrial dysfunction limited subsequent responses to IL-4, whereas restraining nitric oxide-associated respiratory impairment improved repolarization [31]. Mitochondrial injury may therefore limit responses to a change in extracellular signals. Whether this mechanism prevents functional recovery in RA requires direct tests of efferocytosis, barrier support or repair after the inflammatory stimulus is removed.

These findings suggest three distinct aims for target selection. Inhibit demonstrated dependencies that maintain inflammatory output or antigen presentation, including nutrient-stress survival where it sustains pathogenic lymphocyte stimulation. Preserve macrophage populations that demonstrably clear apoptotic cells, support the lining barrier or promote repair. Restore mitochondrial or NAD homeostasis when metabolic injury compromises these functions or promotes inflammatory death [15,20,21,29,31]. These are testable therapeutic priorities, not a validated assignment of every macrophage subset to treatment. Antigen presentation, efferocytosis, barrier integrity, repair and inflammatory cell death should be assessed separately; marker expression cannot substitute for these outcomes.

### 3.2. Metabolic Defects and Adaptation in T Cells

Defined populations of RA T cells show a metabolic defect that differs from the glycolytic activation observed in fibroblasts. In patient-derived naive CD4 T cells studied after activation, insufficient induction of 6-phosphofructo-2-kinase/fructose-2,6-bisphosphatase 3 (PFKFB3) reduced glycolytic activity and adenosine triphosphate (ATP) production and impaired autophagy. Glucose was preferentially redirected towards the pentose phosphate pathway (PPP), with increased reduced nicotinamide adenine dinucleotide phosphate (NADPH) and reduced reactive oxygen species. Thus, reduced glycolysis can coexist with pathogenic immune programming [5].

Subsequent work connected this reductive state to defective oxidant signalling and proarthritogenic effector behaviour. The loss of physiological oxidant signalling suggests that further antioxidant exposure could be detrimental in these cells, even when oxidative stress is increased elsewhere in the joint [32].

Metabolic failure can also support tissue invasion. ATP- and pyruvate-poor RA T cells increased fatty acid synthesis, lipid droplets and the scaffolding protein tyrosine kinase substrate with five SH3 domains (TKS5), facilitating invasive membrane structures [33]. A separate study linked deficiency of succinate-CoA ligase GDP-forming subunit beta (SUCLG2) to reductive TCA metabolism, excess citrate and acetyl-CoA, and tubulin acetylation that stabilized a migratory cytoskeleton [34]. Reduced energy production can therefore coexist with increased lipid synthesis and cytoskeletal acetylation. Rescue of pyruvate availability, enzyme reconstitution or interference with the relevant acetylation machinery reduced pathogenic behaviour in the studied systems.

These observations must be interpreted alongside studies of activated synovial T cells, whose phenotype reflects differentiation, stimulation and exposure to local metabolites. Lactate uptake through SLC5A12 can promote interleukin 17 (IL-17) output, fatty acid synthesis and retention despite reduced glycolytic activity. The combination of reduced glycolysis and increased inflammatory function emphasizes the need to compare cells with matched tissue origin, differentiation state and activation conditions [16].

Mitochondrial insufficiency creates further routes to inflammation. Deficiency of the DNA-repair nuclease MRE11A links defective DNA repair to premature T-cell ageing, while loss of mitochondrial protection permits mitochondrial DNA leakage, inflammasome activation and pyroptosis [35,36]. Reduced mitochondrial aspartate production was also connected to altered NAD metabolism, endoplasmic reticulum expansion and increased transmembrane tumour necrosis factor (TNF) biogenesis in RA T cells [6]. These defects affect organelle communication and inflammatory cell death as well as respiration. Their experimental rescue offers a rationale for therapeutic development, but the studies do not establish clinical efficacy of metabolite supplementation or mitochondrial transfer.

Other activated CD4 T-cell studies report increased metabolic activity. In 2024, autocrine TNF stimulated glycolysis, amino acid uptake, glutamine oxidation and mitochondrial biogenesis through IL-2-inducible T-cell kinase (ITK), protein kinase B (Akt) and mechanistic target of rapamycin (mTOR) signalling. Activated RA cells showed increased TNF, Akt phosphorylation and mitochondrial mass [37]. Activation timing, cytokine exposure and cohort differences may contribute to divergence from the PFKFB3-deficient phenotype. Mitochondrial mass also differs from respiratory efficiency.

The balance between T helper 17 (Th17) and regulatory T (Treg) cells captures only part of adaptive immune pathology. Human RA contains B-cell-supporting peripheral helper T cells, and highly polyfunctional synovial T-cell populations can emerge before clinical disease [38,39]. A 2024 study linked excess succinyl-CoA to bromodomain-containing protein 2 (BRD2) succinylation, reduced transcription of proteasome subunit beta 5 (PSMB5), Hobit accumulation and enhanced tissue-resident memory T-cell differentiation; intervention reduced synovitis in humanized chimeras [40]. The pathway connects mitochondrial metabolites to protein turnover and tissue retention, providing a mechanism of persistence beyond changes in the Th17/Treg ratio. Its contribution to remission after treatment withdrawal has yet to be tested.

### 3.3. Metabolic Requirements of Regulatory T Cells

Foundational evidence for divergent effector and regulatory T-cell metabolism comes largely from non-RA models. Pyruvate dehydrogenase kinase 1 (PDHK1) perturbation linked pyruvate allocation to Th17/Treg differentiation in murine inflammation [41,42]. Oxidative phosphorylation (OXPHOS) does not establish fatty acid oxidation (FAO): genetic loss of carnitine palmitoyltransferase 1A (CPT1A) left several Treg outcomes intact despite etomoxir sensitivity [43]. Respiratory-chain dependence, substrate use and inhibitor off-target effects must therefore be assessed separately.

In a non-RA mouse genetic study, Treg-specific respiratory complex III loss impaired suppression without reducing cell number or forkhead box P3 (Foxp3) expression, and altered metabolite pools and DNA methylation [44]. Thus, lineage markers did not establish competence. Non-RA Foxp3/Toll-like receptor (TLR) experiments likewise showed that increased glucose uptake and anabolic signalling could expand Tregs while reducing suppression [45]. These findings define mechanisms to test in RA rather than a measured metabolic state shared by all RA Tregs.

In non-RA experimental systems, Foxp3 enabled Treg function under low-glucose, high-lactate conditions [46]. Conversely, mechanistic target of rapamycin complex 1 (mTORC1)-dependent anabolic metabolism supported suppressive programmes [47]. These results caution against equating regulatory function with a single low-glycolysis or purely oxidative phenotype.

Migration has requirements distinct from suppression. In non-RA mechanistic systems, glucokinase-dependent glycolysis downstream of phosphoinositide 3-kinase (PI3K) and mTOR complex 2 (mTORC2) supported Treg trafficking; cells that remained suppressive in vitro could fail to reach inflamed tissue [48]. An intervention intended to restore tolerance should therefore measure suppression and tissue access separately.

Direct RA evidence is now available, but its sampling context matters. A 2025 study of patient blood-derived T cells and induced Tregs linked uncoupling protein 2 (UCP2), carnitine palmitoyltransferase 2 (CPT2) and cytotoxic T-lymphocyte-associated protein 4 (CTLA4) trafficking to impaired suppression [49]. This supports an RA-associated mechanism without establishing the fuel requirements of freshly isolated synovial Tregs.

Liu et al. reported extracellular acidification and oxygen-consumption measurements in Tregs isolated from RA synovial fluid. Reduced glycolysis coexisted with increased respiration and impaired migration in a cluster of differentiation 31 (CD31)-associated programme [50]. These joint-derived measurements provide direct RA evidence without establishing uniform respiratory failure across synovial states. Comparison with healthy-donor cells still requires attention to compartment and processing.

A 2026 study combined human synovial evidence of lipid-peroxidation stress with synovial-fluid exposure experiments and arthritis models, implicating ferroptotic stress, respiratory-chain injury and TXK tyrosine kinase signalling [51]. Its oxygen-consumption experiments used collagen-induced arthritis synovial fluid; they should be distinguished from flux measurements in freshly isolated human synovial Tregs. Together, these reports support state- and assay-dependent abnormalities. Matched blood, synovial-fluid and tissue Tregs, measured alongside migration and suppression, would help resolve their differences.

### 3.4. Metabolism of Synovial Fibroblasts

Altered glucose metabolism in RA fibroblast-like synoviocytes (FLS) has been linked to invasion and inflammatory activity. Perturbation of hexokinase 2 (HK2) supported a role in synovial pathology using patient-derived cells and experimental models. These findings support the development of interventions directed at fibroblast metabolism [52,53].

PFKFB3 provides a direct comparison between fibroblast and T-cell metabolism. Its expression was increased in RA fibroblasts, and inhibition or depletion reduced inflammatory mediator production, migration and invasion, with benefit in collagen-induced arthritis (CIA) [54]. TLR2 stimulation also coupled mitochondrial abnormalities to altered bioenergetics in RA fibroblasts, with glycolytic blockade reducing pathogenic outputs [55]. These results contrast with deficient PFKFB3 induction in defined RA blood T cells. Systemic inhibition could therefore suppress a fibroblast dependency while worsening a lymphocyte defect. This potential trade-off requires testing at exposures relevant to both populations.

Glutaminase 1 (GLS1) is another experimentally supported candidate: inhibition or depletion reduced proliferation of RA fibroblast-like synoviocytes. The evidence supports a fibroblast dependency; glutamine requirements in lymphoid aggregates remain less well defined. Whether GLS1 dependence persists under physiological substrate concentrations, hypoxia and concurrent disease-modifying antirheumatic drug (DMARD) treatment remains an important translational question [56].

Biosynthesis is broader than glucose and glutamine catabolism. Choline kinase inhibition altered phosphocholine metabolism and reduced fibroblast migration and apoptosis resistance, with efficacy in experimental arthritis [57]. Lipid peroxide handling can also determine survival: TNF signalling increased cystine uptake and glutathione synthesis in ferroptosis-resistant fibroblast states, and combining TNF blockade with a ferroptosis inducer reduced arthritis in mice [58]. This strategy acts through selective death of susceptible fibroblasts, a mechanism distinct from restoring their metabolism.

Fibroblast heterogeneity changes how these dependencies should be evaluated. Human studies identified disease-associated fibroblast populations, while experimental work separated inflammatory from tissue-damaging stromal functions [59,60]. A cross-tissue atlas further distinguished shared immune-interacting and vascular-interacting fibroblast states across chronic inflammatory diseases [61]. Together with endothelial Notch signalling, these findings suggest that dependencies identified in bulk fibroblast cultures may vary by anatomical position and inflammatory state. Validation in defined subsets would help separate effects on recruitment, invasion and repair.

Immune–stromal signalling can itself alter fibroblast metabolism. Activated helper T cells have been shown to increase fibroblast glycolytic activity, supporting a reciprocal relationship between inflammatory instruction and metabolic demand. Whether this metabolic response persists after T-cell signals are removed remains an important question [62].

### 3.5. Metabolism of B Cells and Plasma Cells

RA synovium contains B cells with inflammatory functions beyond antibody secretion. Programmed cell death protein 1 (PD-1)-positive memory B cells accumulate in inflamed synovium, express inflammatory and costimulatory molecules, and show metabolic features favouring glycolysis under the studied conditions [63]. Hypoxic culture preserved inflammatory outputs, and C-X-C motif chemokine receptor 3 (CXCR3) blockade restricted invasion. PD-1 expression in this population accompanies inflammatory activity, rather than indicating exhaustion. Other synovial B-cell states may have different metabolic requirements.

B cells can additionally regulate the metabolism of neighbouring T cells. Inducible T-cell costimulator ligand (ICOSL)-expressing B cells promoted glucose utilization and inflammatory differentiation in T-cell cocultures; patient studies linked B-cell depletion to diminished metabolic and inflammatory helper responses [64]. Here, the metabolic response occurs in recipient T cells, extending the contribution of B cells beyond their own substrate requirements.

Activation and differentiation alter B-cell metabolism. Antigen stimulation increased glycolysis and respiration, while helper signals prevented later mitochondrial failure [65]. Glycogen synthase kinase 3 (GSK3) restrained growth and protected glucose-limited B cells [66]. Non-RA germinal-centre experiments found regional hypoxia and HIF-dependent antibody changes; another tracing study demonstrated substantial FAO with little glucose oxidation [67,68]. These observations generate hypotheses for RA aggregates, whose organization and nutrient environment differ from conventional germinal centres.

Long-lived plasma-cell survival can depend on mitochondrial pyruvate import in non-RA mechanistic studies [69]. This identifies a candidate vulnerability for investigation, not an established therapeutic target in RA ectopic lymphoid structures. Effects on antibody secretion, antigen presentation and long-term humoral protection may diverge. No quantitative evidence presented here establishes RA B-cell aggregates as major glucose or glutamine sinks; abundance, glycolytic signatures and anatomical clustering do not measure net substrate consumption.

### 3.6. Other Immune Cells and the Vascular and Bone Compartments

Dendritic-cell activation couples metabolic demand to antigen presentation. Experimental TLR studies established glycolytic requirements for maturation and rapid biosynthesis of membranes and secretory organelles through TANK-binding kinase 1 (TBK1), inhibitor of nuclear factor kappa B kinase epsilon (IKKε) and Akt signalling [70,71]. In RA, synovial explant-conditioned medium promoted maturation and glycolytic adaptation in monocyte-derived dendritic cells, while synovial CD1c-positive cells expressed enhanced glycolytic programmes relative to blood [72]. These experiments show that soluble factors from RA tissue can induce metabolic and functional changes in dendritic cells.

Neutrophils connect metabolic activity to autoantigen exposure. RA neutrophils showed enhanced extracellular trap formation, and their traps exposed citrullinated antigens and stimulated inflammatory responses in synovial fibroblasts [73]. In a separate mechanistic study, phorbol 12-myristate 13-acetate (PMA)-induced trap release depended strongly on extracellular glucose and glycolysis [74]. The dependence of neutrophil extracellular trap (NET) formation on glycolysis may vary with the stimulus. Testing RA-relevant triggers alongside antimicrobial responses will be necessary to assess the selectivity of metabolic inhibition.

Metabolic interventions may also affect angiogenesis and bone remodelling. Endothelial PFKFB3-dependent glycolysis supports vessel sprouting and migration in experimental models [75], suggesting a potential link between endothelial metabolism and the vascular–stromal interactions discussed above. In bone, MYC- and estrogen-related receptor alpha (ERRα)-driven oxidative metabolism supported osteoclastogenesis, whereas propionate and butyrate shifted osteoclast metabolism towards glycolysis and reduced differentiation and pathological bone loss in experimental systems [76,77]. In these osteoclast models, increased glycolysis accompanied reduced bone resorption. ACOD1-related pathways also regulate inflammatory bone outcomes, as discussed below [78]. Effects on inflammation, erosion and bone formation may therefore differ.

**Figure 1:**
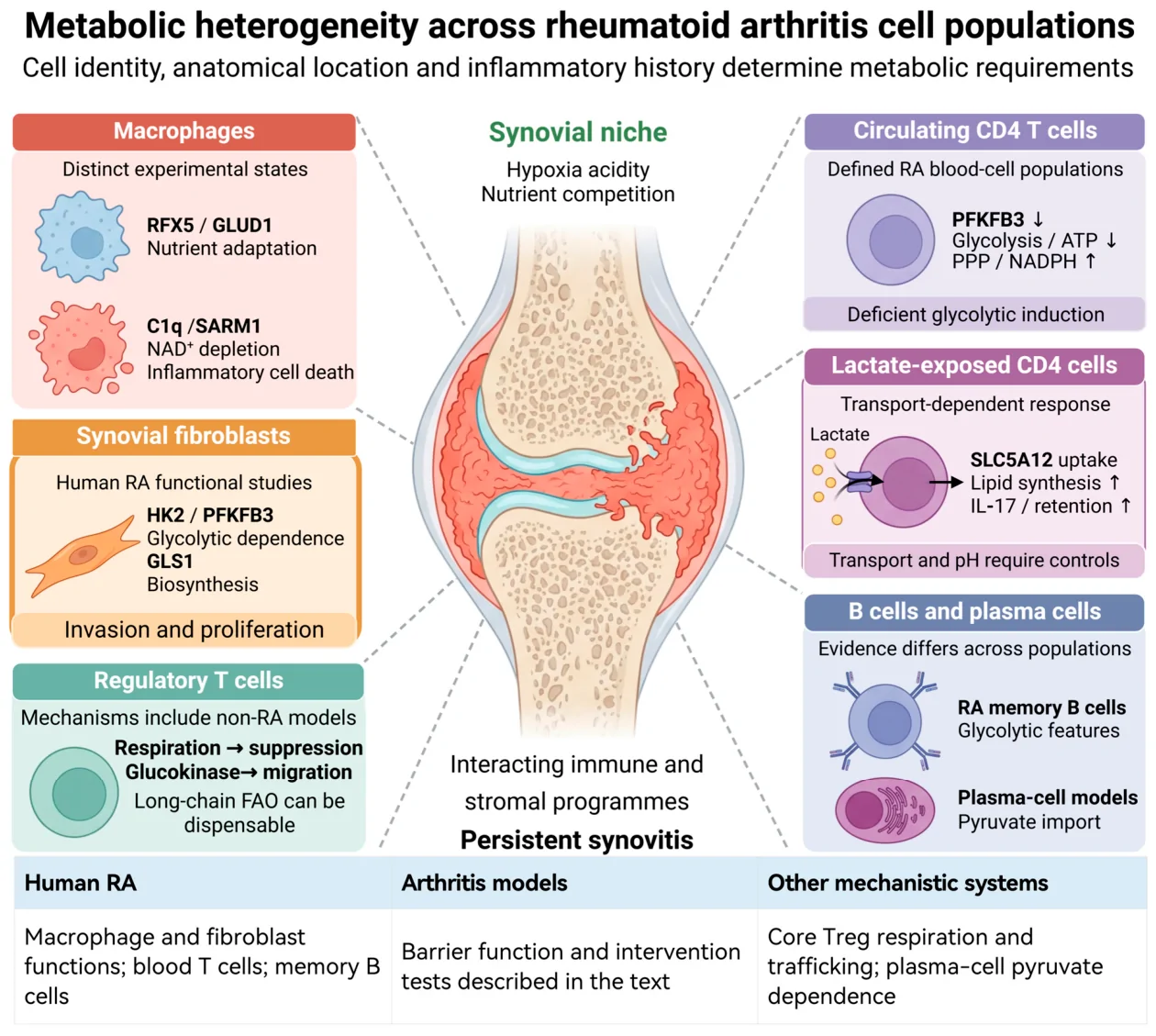
Metabolic heterogeneity across rheumatoid arthritis cell populations. The joint cutaway is schematic and does not map measured oxygen, pH or nutrient gradients. Macrophage nutrient adaptation and metabolic exhaustion describe distinct experimental states. Fibroblast dependencies and the PFKFB3-deficient blood T-cell phenotype are supported by human RA studies. The Treg respiration and glucokinase examples shown are foundational non-RA mechanisms; newer RA blood-, fluid- and tissue-associated findings are discussed separately in Section 3.3. Plasma-cell pyruvate dependence is a non-RA extrapolation. Dashed lines organize the populations and do not demonstrate metabolite transfer. RA, rheumatoid arthritis; RFX5, regulatory factor X5; GLUD1, glutamate dehydrogenase 1; C1q, complement component 1q; SARM1, sterile alpha and Toll/interleukin-1 receptor motif-containing 1; NAD, nicotinamide adenine dinucleotide; HK2, hexokinase 2; PFKFB3, 6-phosphofructo-2-kinase/fructose-2,6-bisphosphatase 3; GLS1, glutaminase 1; CD4, cluster of differentiation 4; ATP, adenosine triphosphate; PPP, pentose phosphate pathway; NADPH, reduced nicotinamide adenine dinucleotide phosphate; SLC5A12, solute carrier family 5 member 12; IL-17, interleukin 17; FAO, fatty acid oxidation. See Section 3 and Table 1. (Upward (↑) and downward (↓) arrows denote increases and decreases in the labelled parameters, respectively, relative to the comparators used in the cited studies. Grey dashed lines connect the cell-population panels to the central joint schematic for illustration and do not indicate cell migration or causal relationships.)

Figure 1 and Table 1 distinguish the source and functional meaning of selected metabolic findings. Target selection requires a demonstrated dependency in a defined state, together with assessment of regulatory, reparative and host-defence functions exposed to the same intervention.

## 4. Metabolite Signalling and Inflammatory Persistence

### 4.1. Lactate Transport and Extracellular pH

Lactate can act as a substrate, a transport-dependent signal and a contributor to extracellular acidity, with different consequences for different cells. Early RA-relevant experiments distinguished sodium lactate-associated effects on CD4 T-cell motility from lactic-acid effects on cluster of differentiation 8 (CD8) T cells, involving different transporters and metabolic responses [79]. Subsequent work implicated SLC5A12-dependent uptake in CD4 T-cell retention, lipid synthesis and IL-17 production [16]. These experiments connect a specific transporter to changes in T-cell migration and inflammatory function.

Lactate can also support regulatory activity. In tumour models, monocarboxylate transporter 1 (MCT1)-dependent lactate uptake sustained intratumoral Treg function, extending the Foxp3-associated adaptation observed in foundational studies [46,80]. Although derived from non-RA systems, these findings identify a possible cost of lactate-transport inhibition: loss of a substrate supporting Treg suppression. The balance will depend on transporter expression and function in the populations exposed to treatment.

Extracellular pH provides a separate source of variation. Lactate concentration reflects production, transport and clearance, whereas acidity also depends on ATP turnover and buffering. Experiments using sodium lactate therefore need controls for pH and osmolarity. Sodium-coupled SLC5A12 uptake and proton-coupled MCT1 and monocarboxylate transporter 4 (MCT4) transport may produce different responses even at similar lactate concentrations.

A 2025 study connected lactate signalling to ectopic lymphoid organization and showed pro-resolution effects of SLC5A12 blockade. Its principal disease context was Sjögren’s disease and experimental salivary-gland inflammation, rather than RA. The findings support testing whether similar transport-dependent interactions sustain synovial lymphoid aggregates [81]. Their relevance to RA will depend on direct evidence of this interaction in synovial tissue.

### 4.2. Succinate Sensing and Receptor Localization

Macrophage experiments linked succinate accumulation to HIF-1α-dependent IL-1β regulation [24]. An RA-relevant study subsequently demonstrated that extracellular succinate, acting through succinate receptor 1 (SUCNR1, also known as GPR91), contributed to macrophage activation; receptor deficiency reduced disease in antigen-induced arthritis, and RA synovial fluids elicited receptor-dependent effects in macrophage assays [82]. Intracellular succinate and extracellular SUCNR1 signalling are related but separately measurable processes.

SUCNR1 signalling also links the joint to lymphoid priming. Initial dendritic-cell studies showed succinate-dependent migration and enhanced antigen-specific immune responses [83]. In antigen-induced arthritis, receptor deficiency reduced dendritic-cell trafficking and Th17 expansion in draining lymph nodes; adoptive-transfer experiments supported a dendritic-cell contribution [84]. SUCNR1 modulation may therefore affect lymphoid priming as well as local macrophage activity.

A 2026 report linked concentration-dependent succinate responses to SUCNR1 internalization and mitochondrial redistribution in RA-associated macrophage experiments [85]. This is a recent single-study mechanism for which independent replication has not yet been established in the literature assessed here. Its therapeutic implications cannot be generalized to all RA macrophage states. Confirmation should distinguish plasma-membrane from mitochondrial receptor pools, use compartment- and receptor-specific controls, and test inflammatory and resolving populations separately.

Bulk succinate should be paired with receptor localization and recipient-cell response. A fall in circulating succinate may reflect systemic change without engaging a synovial circuit; receptor modulation may alter function without normalizing the metabolite pool. These are different pharmacodynamic questions.

### 4.3. Itaconate and Lipid Metabolism

ACOD1 generates itaconate during inflammatory metabolic remodelling. Genetic studies linked endogenous itaconate to succinate dehydrogenase inhibition and altered macrophage respiration and cytokine production [86]. Work on itaconate and electrophilic derivatives subsequently identified Kelch-like ECH-associated protein 1 (KEAP1)–nuclear factor erythroid 2-related factor 2 (NRF2) and inhibitor of nuclear factor kappa B zeta (IκBζ)–activating transcription factor 3 (ATF3) mechanisms [87,88]. Additional studies implicated NLR family pyrin domain-containing 3 (NLRP3) modification and tolerance to late inflammasome activation [89,90]. The effects involve both metabolic enzymes and inflammatory signalling, with timing and chemical form influencing the response.

Arthritis-model studies extend itaconate biology to fibroblasts and bone. Native itaconate reduced fibroblast proliferation, migration and bioenergetic activity, and improved rat arthritis after intra-articular delivery [91]. Bone findings diverge: one study found greater osteoclastogenesis and erosion after Acod1 loss; another found the endogenous axis dispensable for osteoclast generation and implicated osteoblast differentiation and bone formation [78,92]. Cell preparation, model and endogenous versus exogenous exposure may contribute. Resorption and formation require separate endpoints.

Endogenous itaconate, dimethyl itaconate and 4-octyl itaconate also differ in uptake, electrophilicity and biological activity [93]. A derivative’s effect therefore cannot be assigned automatically to the endogenous metabolite. Moreover, a 2025 study identified pro-inflammatory itaconate activity in resident alveolar macrophages during acute lung injury [94]. Although its relevance to RA remains uncertain, this result indicates that itaconate can have different effects in tissue-resident macrophages.

Lipid synthesis, storage and oxidation support different cellular functions. Acetyl-CoA carboxylase 1 (ACC1)-dependent de novo fatty acid synthesis supported Th17 differentiation relative to Treg generation in experimental studies [95], whereas lysosomal lipolysis supported selected IL-4-induced macrophage functions [96]. Genetic work on long-chain fatty acid oxidation and studies of etomoxir off-target effects challenged the inference that all such functions require mitochondrial FAO [97,98]. Lipid droplets or acylcarnitines likewise cannot identify a particular flux without tracing or functional perturbation.

In RA, lipid metabolism may support membrane synthesis for invasion, mitochondrial substrate use or resistance to lipid peroxidation. The TKS5 and ferroptosis studies illustrate how lipid-associated pathways can support different pathogenic functions [33,58]. The functional effects of inhibition will depend on which of these processes is disrupted.

### 4.4. Amino Acid Metabolism

Glutamine supports carbon and nitrogen metabolism, but transporter activity and downstream enzymatic steps need not have equivalent effects. Alanine-serine-cysteine transporter 2 (ASCT2)-dependent uptake supported mTORC1 activation and inflammatory T-cell responses in experimental models [99]. In contrast, glutaminase (GLS) deficiency impaired Th17 differentiation while initially enhancing aspects of T helper 1 (Th1) and cytotoxic T-cell function, demonstrating lineage-specific consequences of a downstream intervention [100]. RA fibroblast GLS1 dependence and macrophage GLUD1-mediated adaptation therefore warrant investigation as separate therapeutic targets [15,56].

Arginine metabolism further separates persistence from inflammatory output. Increasing intracellular arginine promoted oxidative metabolism, survival and central-memory-like properties in activated T cells in experimental studies [101]. In a distinct study, modelling followed by genetic and chemical perturbation implicated downstream polyamine metabolism in Th17 pathogenicity [102]. Circulating amino acid concentrations provide limited information about these intracellular pathways. Interpretation requires evidence of substrate use in the relevant cell population.

The indoleamine 2,3-dioxygenase (IDO)–kynurenine–aryl hydrocarbon receptor (AHR) pathway has both regulatory and inflammatory effects. Experimental studies showed ligand-dependent promotion of regulatory or Th17 programmes through AHR, and kynurenine supported regulatory T-cell generation in a defined experimental setting [103,104,105]. In collagen-induced arthritis, pharmacological IDO inhibition worsened disease, while genetic deficiency and kynurenine administration supported a counter-regulatory role for tryptophan catabolism [106,107]. An increased kynurenine/tryptophan ratio can therefore reflect feedback regulation rather than a pathogenic signal requiring inhibition.

The opposite therapeutic direction was observed in the K/BxN system: pharmacological IDO inhibition constrained autoreactive B-cell differentiation and recurrence after B-cell repopulation, and combination with methotrexate improved arthritis [108,109]. This comparison illustrates context dependence rather than a uniform recommendation to inhibit or enhance tryptophan catabolism. Disease model, dominant effector cells, disease stage, inhibitor and isoform selectivity, and background treatment can all affect interpretation. A kynurenine/tryptophan ratio alone cannot identify the responding cell or guide treatment; cell-resolved functional and pharmacological evidence is needed.

### 4.5. Metabolic Regulation of Chromatin and Cellular Memory

Metabolites influence chromatin through substrate supply, enzyme cofactors and signalling. ATP-citrate lyase (ACLY) connected glucose-derived citrate to histone acetylation in mammalian-cell experiments [110]. In macrophages, Akt–mTORC1–ACLY signalling regulated selected IL-4-associated genes, while glutaminolysis-derived α-ketoglutarate supported lysine demethylase 6B (Jmjd3)-dependent chromatin regulation [111,112]. These mechanisms establish biochemical routes from metabolism to transcription, but their effect depends on locus, cell state and competing metabolites.

Trained-immunity studies show that metabolism can influence later responses. Human monocyte experiments connected beta-glucan training to glycolysis and chromatin states [113,114]; subsequent perturbations implicated glutaminolysis, fumarate and mevalonate [115,116]. These defined training and rechallenge protocols establish candidate mechanisms, not a demonstrated cause of persistent RA autoimmunity.

Human RA fibroblasts exhibit disease-associated DNA methylation and integrated epigenomic features, including differences between anatomical joint sites [117,118,119]. These signatures are consistent with persistent stromal specialization, although their origin may involve positional identity or inflammatory exposure as well as metabolism. Longitudinal perturbation would help determine which marks depend on metabolism and whether they persist within surviving fibroblasts.

Following the identification of histone lactylation, studies have examined its contribution to RA fibroblast activation [120]. In 2025, a study linked histone H3 lysine 9 (H3K9) lactylation in patient-derived fibroblasts to nuclear factor of activated T cells c2 (NFATc2) expression and invasive behaviour, with functional and arthritis-model experiments supporting the pathway [121]. Anti-lactylated histone antibodies were also associated with disease activity, but their diagnostic or predictive value remains exploratory. A separate study implicated histone H3 lysine 18 (H3K18) lactylation and sirtuin 3 (SIRT3)-associated delactylation in fibroblast activation and macrophage–fibroblast interactions [122]. H3K9 and H3K18 lactylation therefore need to be assessed as distinct modifications with potentially different regulatory effects.

Together with BRD2 succinylation in RA T cells [40], these studies extend metabolic regulation beyond energy production. Because lactate metabolism and deacylases affect several pathways, a memory mechanism requires modification-specific perturbation and rescue, followed by persistence testing after washout.

## 5. Metabolic Checkpoints in Effector and Regulatory Cells

### 5.1. HIF-1α in Hypoxic and Inflammatory Responses

HIF-1α coordinates responses to low oxygen with inflammatory and metabolic signals. Myeloid-specific deletion established requirements for glycolytic capacity, motility and inflammatory function in experimental models [123]. In T-cell differentiation experiments, HIF-1 promoted retinoic acid receptor-related orphan receptor gamma t (RORγt)-associated Th17 programmes and favoured Foxp3 degradation [124]. However, studies of inflammatory mucosal hypoxia found HIF-1α-dependent Foxp3 induction and a requirement for Treg-mediated protection [125]. These results identify both effector and regulatory functions of HIF-1α. Its effects in RA will depend on which populations are exposed to inhibition and how they respond under synovial conditions.

HIF activity should be interpreted alongside oxygen, respiration and viability. Reduced inflammatory transcription can accompany failed hypoxic adaptation or cell loss, so these outcomes should be measured separately.

### 5.2. mTOR Signalling in Effector and Regulatory Cells

mTOR integrates nutrient and growth signals through mTORC1 and mTORC2. Genetic deletion studies separated effector from regulatory lineage commitment and identified distinct complex-dependent requirements for helper T-cell differentiation [126,127]. Measurements of mTOR activity therefore need to identify the complex and downstream substrate being assessed. The same phosphoprotein readout may have different functional implications across synovial populations.

mTOR-supported biosynthesis can sustain effector expansion, yet mTORC1 also supports Treg suppression [47]. Complete blockade and timed partial inhibition may therefore produce different outcomes. Dose and tissue exposure should be linked to effector activity and regulatory function in parallel.

### 5.3. AMPK Localization and Energy Sensing

RA T-cell studies identified impaired N-myristoyltransferase-1 (NMT1) activity as a mechanism disrupting AMP-activated protein kinase (AMPK) localization and activation, permitting excessive mTORC1 signalling. This identifies a specific defect in energy sensing in RA T cells [128]. The finding also explains why protein abundance alone may miss impaired AMPK signalling.

AMPK can also preserve inflammatory effector competence under nutrient stress. In experimental T-cell studies, AMPKα1 supported glutamine-dependent mitochondrial adaptation, viability and responses to pathogenic challenge [129]. In a 2024 arthritis-model study, the liver X receptor (LXR) inverse agonist SR9243 altered macrophage glycolytic programmes with accompanying AMPK–mTOR–HIF-1α signalling changes [130]. Because SR9243 acts on LXR, the associated pathway changes do not isolate AMPK as the cause of benefit. Metformin also has multiple effects. The contribution of AMPK to these responses therefore requires target-specific tests.

**Figure 2:**
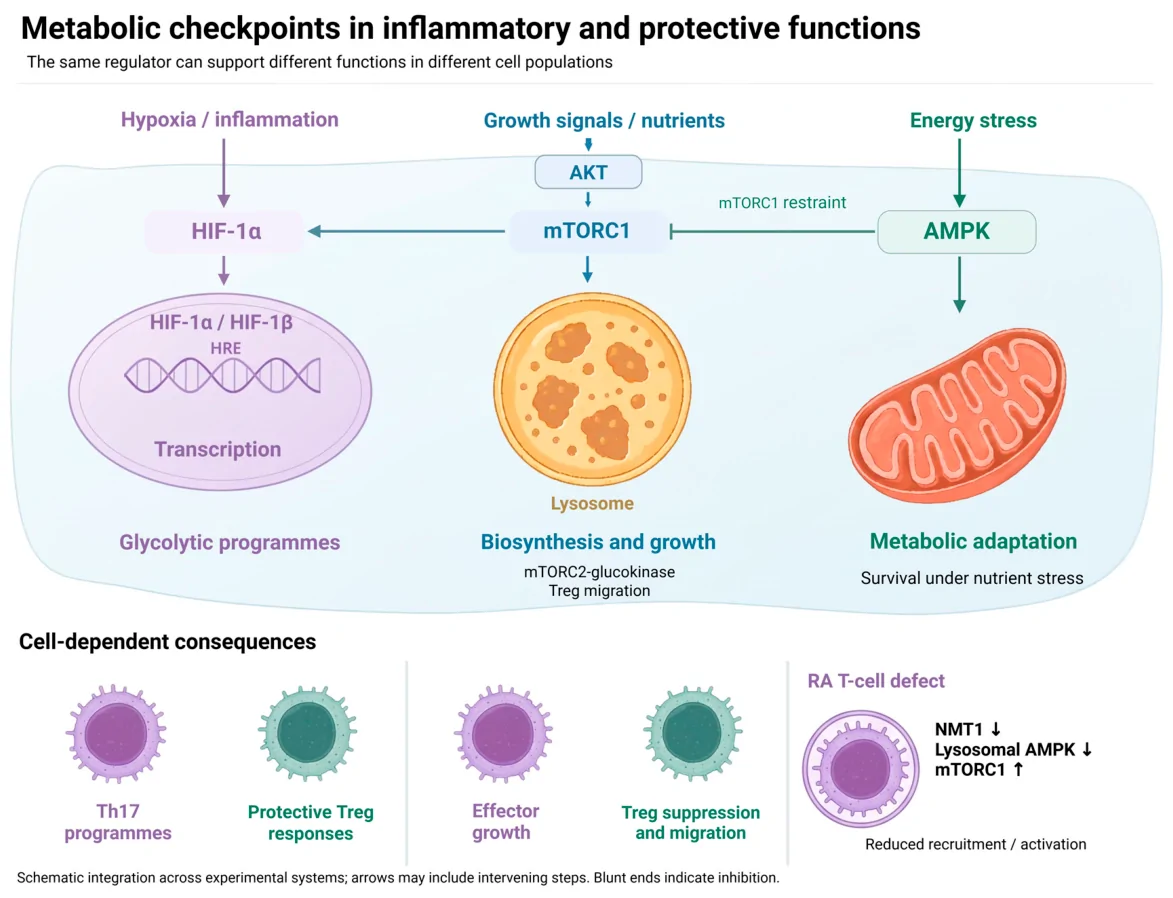
Metabolic checkpoints in inflammatory and protective functions. The upper compartment integrates different experimental systems and is not one pathway verified in a single RA cell state. Lower examples represent distinct populations and contexts. The NMT1–AMPK defect is supported by human RA blood T-cell experiments. Arrows can include intervening steps; blunt ends denote inhibition. RA, rheumatoid arthritis; Treg, regulatory T cell; Th17, T helper 17 cells; Akt, protein kinase B; HIF, hypoxia-inducible factor; HIF-1α and HIF-1β, HIF-1 alpha and beta subunits; HRE, hypoxia-response element; mTOR, mechanistic target of rapamycin; mTORC1 and mTORC2, mTOR complexes 1 and 2; AMPK, AMP-activated protein kinase; NMT1, N-myristoyltransferase 1. Section 5 provides the source-specific interpretation. (Upward (↑) and downward (↓) arrows denote increases and decreases in the labelled parameters, respectively, relative to the comparators used in the cited studies.)

Figure 2 integrates HIF-1α, mTOR and AMPK signalling with their context-dependent functional outcomes. For each pathway, the relevant therapeutic question is which function changes in the exposed RA cell population, and whether that change preserves protective activity.

Evidence from different systems is combined here; this is not one pathway verified in a single RA cell state.

## 6. Metabolic Interventions and Therapeutic Selectivity

### 6.1. Selective Metabolic Inhibition

Patient-derived fibroblast experiments and arthritis models support HK2, PFKFB3 and GLS1 as preclinical candidates [53,54,56]. They do not establish clinical benefit from selectively targeting these enzymes in RA. A dependency must persist at achievable exposure and in the presence of standard treatment. The PFKFB3 contrast is particularly consequential: inhibiting a fibroblast dependency could aggravate deficient glycolytic induction in defined blood T-cell states [5,54].

SLC5A12 and SUCNR1 likewise remain preclinical candidates in the studies reviewed [16,82,84,85]. Transporter selectivity, receptor location and the exposed recipient state determine which function may change. Tissue exposure and functional target engagement are needed before extrapolating to clinical efficacy.

Compensatory substrate use may preserve inflammation despite biochemical inhibition. Combination studies should identify that route under physiological nutrient conditions and distinguish reduced pathogenic output from cell depletion or generalized loss of protein synthesis.

### 6.2. Restoring Metabolic Function and Repurposing Drugs

Restoring a deficient function may be as rational as inhibiting an excessive one. RA T-cell studies support experimental rescue of mitochondrial protection, pyruvate supply and organelle communication, while the SARM1 study identifies preservation of macrophage NAD metabolism as a way to limit inflammatory cell death [6,29,33,36]. Each approach addresses a defined defect. Delivery to the relevant population, persistence of the response and effects on other cells remain central development questions.

Metformin has been evaluated as an adjunct to conventional therapy in small clinical trials. A double-blind, placebo-controlled trial randomized 120 patients to metformin or placebo alongside methotrexate for 12 weeks and reported improved short-term clinical responses [131]. The methotrexate dose was 7.5 mg per week, limiting inference about incremental benefit alongside more intensively optimized regimens. A separate single-blind trial randomized 66 patients on stable conventional synthetic DMARDs to metformin or placebo for six months; 60 completed follow-up, with improved inflammatory and clinical measures and three metformin withdrawals for gastrointestinal intolerance [132]. The results justify further trials with optimized background therapy and longer follow-up. Effects on structural progression and disease control after withdrawal remain untested.

Clinical studies of mTOR inhibition have reported efficacy signals alongside tolerability concerns. A 121-patient, double-blind trial of everolimus with methotrexate reported a higher 12-week response rate using the American College of Rheumatology 20% improvement criteria (ACR20) than placebo (36.1% versus 16.7%), alongside more gastrointestinal, skin and laboratory adverse events [133]. A 62-patient, open-label randomized study of low-dose sirolimus over 24 weeks reported improved disease activity and increased circulating Treg numbers [134]. These studies assessed clinical responses during treatment; they did not establish restored tissue Treg suppression or persistence after withdrawal. Further development will depend on the balance between incremental benefit and toxicity.

Three claims should be separated when interpreting these trials. Clinical efficacy is improvement in a prespecified patient outcome. Mechanistic evidence connects a biological change to a proposed pathway. Metabolic target engagement requires evidence that achievable exposure alters the intended reaction, transport process or signalling event in the intended cells. Serum cytokines, AMPK transcript abundance or circulating Treg counts may support mechanistic hypotheses, but do not demonstrate correction of synovial metabolic flux or suppressive function [131,132,133,134]. These exploratory trials therefore support on-treatment efficacy signals, not a clinically established cell-selective metabolic mechanism or durable reprogramming.

### 6.3. Combination Therapy and Local Delivery

Established RA drugs also act on metabolic processes. Early experiments showed that methotrexate enhanced adenosine release from connective-tissue cells and reduced neutrophil adherence [135]. Cytokine blockade can also alter the metabolic instructions received by lymphocytes and fibroblasts [37,58]. Candidate combinations need to be evaluated alongside effective DMARD therapy, with attention to overlapping metabolic effects. The ferroptosis/TNF-blockade and IDO/methotrexate studies illustrate potentially useful interactions that remain to be evaluated clinically.

Local delivery offers experimental control of exposure. Intra-articular native itaconate improved arthritis in rats [91]. A 2025 study used near-infrared-responsive nanoparticles to control haem oxygenase 1 (HO-1) gene expression and alter synovial-cell metabolism in collagen-induced arthritis [136]. Both approaches remain experimental. Joint retention, cell-specific uptake, systemic escape and the contribution of photothermal or carrier effects require measurement. Accumulation in an inflamed joint may still expose several cell populations, and repeated local treatment may be impractical in polyarticular disease.

### 6.4. Nutritional Strategies and Metabolic Exposures

Nutrition provides an additional route to modify systemic metabolic exposures. The 16-week Plants for Joints trial randomized 83 patients with RA to a programme combining a whole-food plant-based diet, physical activity and stress management or usual care, and reported improved disease activity and metabolic measures [137]. Because this was a multicomponent intervention, neither diet alone nor a specific synovial metabolic mechanism can be assigned the benefit.

Conforti et al.’s 2026 scoping review distinguishes ketogenic protocols with nutritional ketosis from fasting or low-carbohydrate interventions without verified ketosis [138]. Its strongest human signals concern psoriatic disease; RA evidence remains largely mechanistic or ketosis-adjacent. This supports investigation, rather than an efficacy claim for ketogenic diets in RA. In primary non-RA experiments, beta-hydroxybutyrate (BHB) inhibited NLRP3 inflammasome activation [139]. A subsequent study demonstrated pH-dependent effects in mouse macrophages and human peripheral blood cells, reinforcing the need to control formulation and acidity [140]. These experiments do not show that dietary ketosis achieves the same exposure or effects in RA synovium.

Future RA trials should verify ketosis when relevant, use an active dietary comparator, quantify energy intake, weight and body composition, and maintain interpretable background DMARD therapy. Joint outcomes should be evaluated alongside lipids, glycemic status, tolerability and nutritional adequacy. Weight-related improvement, systemic metabolic change and cell-specific immunomodulation should be analyzed as distinct hypotheses. Nutrition is considered here as an adjunct to established treatment; the current evidence does not establish withdrawal of DMARDs or durable cellular reprogramming.

Table 2 separates target-specific preclinical approaches, exploratory drug trials and nutritional or lifestyle interventions. Clinical improvement is not proof of a proposed synovial mechanism. Translation requires linking exposure and metabolic target engagement to function in the intended cells.

## 7. Translational Development and Sustained Treatment Effects

### 7.1. Selecting Patients for Metabolic Interventions

Synovial pathotypes can support stratification but do not establish metabolic drug sensitivity. Oxygenation, medication, cardiometabolic comorbidity and cellular composition may differ within the same category. Selection should therefore combine the proposed tissue mechanism with systemic covariates and, where feasible, an ex vivo functional response.

Biopsy-driven biologic trials illustrate both the promise and the limitations of this approach. The Rituximab versus Tocilizumab in Anti-TNF Inadequate Responder Patients with RA (R4RA) trial demonstrated the feasibility of tissue-based clinical investigation, and subsequent molecular analyses linked synovial features to differential treatment outcomes [141,142]. However, Stratification of Biological Therapies by Pathobiology (STRAP) and STRAP-EU did not establish baseline B-cell status as a predictor of response to B-cell depletion in their biologic-naive population [143]. The results show that a plausible tissue classifier may fail to predict treatment benefit in a new population.

The 2025 STRAP molecular-profiling study identified additional response-associated signatures and predictive models, supporting continued development of tissue-guided treatment selection [144]. Their performance needs to be assessed for the intended population, assay and outcome, and cannot be transferred directly to metabolic module scores. For a metabolic agent, a prospective trial should test the treatment-by-biomarker interaction; an association between a biomarker and improvement within the treated group alone is insufficient.

### 7.2. Measuring Target Engagement and Functional Response

An early-phase programme should connect drug exposure in the relevant compartment with a biochemical or signalling effect and a defined cellular function. For example, a transport inhibitor should show altered transport or downstream response, together with a change in the intended inflammatory behaviour. Transporter abundance identifies where a drug might act; altered transport or signalling provides evidence that it has acted. Metabolite measurements and transcriptional responses can then help interpret the functional outcome.

Metabolite pool size reflects the balance of synthesis, consumption and transport; isotope tracing can resolve carbon contributions, while quantitative flux estimation requires additional measurements and model assumptions [17,18]. Oxygen consumption and extracellular acidification provide complementary functional information but do not, alone, identify every supporting substrate. Viability, cell size and cellular composition also need to be accounted for when comparing metabolic measurements.

Single-cell methods measure different properties. Single-cell energetic metabolism by profiling translation inhibition (SCENITH) infers energetic dependencies from translation responses to inhibitors; Met-Flow measures metabolic-protein abundance alongside phenotype [145,146]. Compass uses transcriptomes and constrained modelling to generate metabolic hypotheses, with experimental examples in Th17 biology [102]. These measurements can guide perturbation, but none is an interchangeable measure of direct metabolic flux.

Spatial metabolomics can complement these approaches by mapping metabolite-associated signals to histologically defined niches, provided annotation confidence and ionization effects are addressed [147]. It measures spatial abundance, not production rate or transport direction. Stable-isotope tracing in defined cells, explants or appropriately designed in vivo studies can test substrate contribution, while extracellular uptake and release rates and kinetic modelling strengthen flux inference [17,18]. Co-registration with cell markers and paired functional perturbation is needed to connect a spatial signal to a cellular dependency.

Culture conditions can expose or obscure a dependency. Human plasma-like medium alters substrate use, redox state and drug sensitivity [148]. RA experiments should report nutrient concentrations, oxygen, serum, stimulation duration and processing conditions. Serial analysis must distinguish within-cell change from selective loss or a changed population mixture.

### 7.3. Testing the Persistence of Metabolic Reprogramming

We propose that durable reprogramming requires a functional change in surviving cells that outlasts pharmacologically relevant exposure. Studies should prespecify the population, functional endpoint and follow-up relative to drug clearance and cell turnover. After non-cytotoxic treatment and demonstrated target engagement, washout should be verified by drug or active-metabolite measurement, or by a justified exposure and pharmacodynamic assessment. The same defined population should then undergo standardized rechallenge and serial functional testing. Residual drug, selective depletion, cell replacement and changed population composition must be excluded as sufficient explanations.

Persistence should be assessed together with competence. Depending on the population, endpoints could include regulatory suppression and migration, efferocytosis, barrier support, repair or responses to an unrelated microbial stimulus. Persistent suppression of all responses would suggest sustained impairment rather than selective correction. A lasting change in a metabolite, transcript or chromatin mark alone is insufficient; linking it to persistent function requires perturbation and rescue. These are proposed evaluation criteria, not evidence that existing RA metabolic interventions meet them.

Clinical evaluation must test whether persistent cellular change contributes to sustained disease control. Paired tissue analysis, imaging, structural outcomes and patient-reported measures can address effects missed by an activity score. Any tapering or withdrawal requires a prespecified trial strategy with flare monitoring and rescue treatment. Remission alone does not identify a metabolic mechanism, and a metabolic biomarker does not establish that reducing treatment is safe.

Clinical withdrawal studies show why on-treatment control is an insufficient surrogate. In RETRO, remission was less frequently maintained after tapering or stopping DMARDs than after continuation; ARCTIC REWIND likewise found more flares with half-dose than stable-dose conventional therapy [149,150]. These trials establish the importance of prospective follow-up when treatment is reduced, although they do not identify a metabolic cause of relapse.

**Figure 3:**
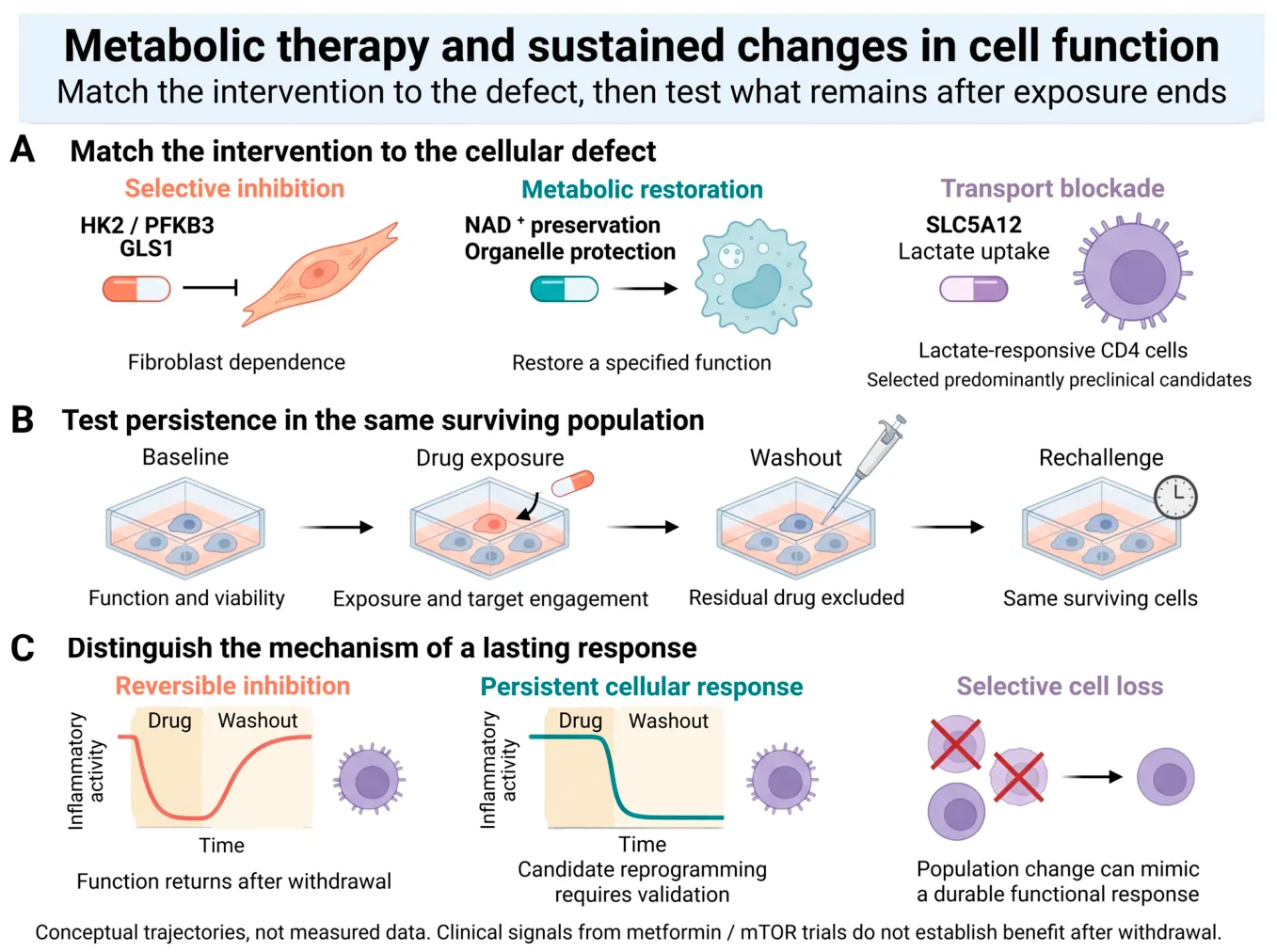
Metabolic therapy and sustained changes in cell function. (**A**) Representative predominantly preclinical interventions illustrate inhibition of fibroblast dependencies, restoration of deficient organelle or NAD metabolism, and blockade of SLC5A12-mediated lactate uptake in responsive CD4 T cells. (**B**) Baseline function and viability are followed by drug exposure and target-engagement measurements, washout with exclusion of residual drug, and standardized rechallenge in the same surviving population. (**C**) Conceptual trajectories use inflammatory activity as an illustrative readout to distinguish a reversible effect from a persistent response requiring mechanistic validation. The curves contain no measured data or effect-size estimates. Selective loss of pathogenic cells can change bulk functional readouts without reprogramming surviving cells. A persistent response therefore requires controls for viability and composition and does not alone establish epigenetic reprogramming. Exploratory RA trials of metformin and mTOR inhibitors provide on-treatment clinical signals; benefit after withdrawal remains unestablished. This framework is a proposed research sequence, not a validated clinical treatment-selection or withdrawal algorithm. The rationale and evaluation criteria are discussed in Section 6 and Section 7. RA, rheumatoid arthritis; CD4, cluster of differentiation 4; HK2, hexokinase 2; PFKFB3, 6-phosphofructo-2-kinase/fructose-2,6-bisphosphatase 3; GLS1, glutaminase 1; SLC5A12, solute carrier family 5 member 12; NAD, nicotinamide adenine dinucleotide; AMPK, AMP-activated protein kinase; mTOR, mechanistic target of rapamycin.

Figure 3 links representative intervention strategies (panel A) to an exposure–washout–rechallenge sequence (panel B) and the alternative explanations for a lasting response (panel C). An effect that disappears after washout supports reversible inhibition, which may still be therapeutically useful. Improvement caused by selective loss of pathogenic cells supports a depletion mechanism. A claim of reprogramming within surviving cells requires evidence that their function remains altered after exposure has ended.

## 8. Conclusions

RA involves metabolic abnormalities that differ across cell populations and anatomical compartments. The clearest therapeutic implications arise where patient-derived functional studies link a specific defect to invasion, antigen presentation or impaired immune regulation. Synovial atlases can help locate these populations, while selective perturbation is needed to establish their metabolic requirements. The contrast between glycolytic fibroblasts and metabolically deficient T cells illustrates why systemic inhibition may produce competing effects in the same disease.

The immediate priority is to establish an exposure that suppresses pathology while preserving regulation, repair and host defence. Persistence after withdrawal is a separate question. Paired tissue and systemic measurements, including cardiometabolic covariates, should connect target engagement to added benefit alongside established RA treatment. Nutritional strategies and repurposed drugs warrant further evaluation without treating preliminary clinical signals as proof of durable reprogramming.

## Figures and Tables

**Table 1:** Metabolic findings across cell populations in rheumatoid arthritis.

Population	Selected Evidence	Limitations	Functional Outcomes
RA fibroblasts	Human RA cells and arthritis models: HK2/PFKFB3/GLS1 and choline metabolism [53,54,56,57]	Subset and culture dependence	Invasion, inflammatory mediators and survival
Defined blood CD4 T cells	Human RA blood T cells: PFKFB3 deficiency, reductive stress and MRE11A/SUCLG2 defects [5,32,34,36]	Activation, cohort and cell-state differences	Redox balance, organelle integrity and invasion
Tissue-exposed T cells	Human RA and experimental systems: SLC5A12 lactate responses and succinyl-CoA/BRD2/PSMB5 axis [16,40]	Retention differs from durable drug-free remission	Migration, cytokines and tissue retention
Inflammatory macrophages	Human RA macrophages: RFX5/GLUD1 nutrient adaptation and antigen presentation [15]	Requirements vary across macrophage states	Survival under nutrient stress and T-cell stimulation
Repair-associated macrophages	Human RA and experimental systems: remission-associated MerTK states; C1q/SARM1-associated death [21,29]	Repair-associated markers can persist despite loss of function	Efferocytosis, tissue repair and inflammatory cell death
Regulatory T cells	Complex III and glucokinase: non-RA mechanisms [43,44,48]; emerging RA measurements [49,50,51]	Blood-derived, synovial-fluid and model findings must be distinguished	Suppression, migration and lineage stability
B cells and plasma cells	Human RA B-cell studies [63,64]; non-RA plasma-cell mitochondrial mechanism [69]	RA B-cell findings versus non-RA plasma-cell mechanism; no quantified nutrient sink	Antibody secretion, antigen presentation and helper activity
DCs and neutrophils	Human RA-relevant DC/NET studies [72,73]; non-RA glycolysis experiments [74]	Culture and trigger dependence	Antigen presentation, NET release and host defence
Vascular and bone cells	Non-RA mechanistic and arthritis-model evidence: endothelial PFKFB3; osteoclast metabolism and SCFA responses [75,76,77]	Mechanisms include non-RA models	Vessel sprouting, erosion and bone formation

Evidence origin is identified in each row as human RA, arthritis model or non-RA mechanism; these are source categories, not formal quality grades. Function is assessed separately from expression. RA, rheumatoid arthritis; CD4, cluster of differentiation 4; HK2, hexokinase 2; PFKFB3, 6-phosphofructo-2-kinase/fructose-2,6-bisphosphatase 3; GLS1, glutaminase 1; MRE11A, DNA-repair nuclease; SUCLG2, succinate-CoA ligase GDP-forming subunit beta; SLC5A12, solute carrier family 5 member 12; BRD2, bromodomain-containing protein 2; PSMB5, proteasome subunit beta 5; RFX5, regulatory factor X5; GLUD1, glutamate dehydrogenase 1; MerTK, MER tyrosine kinase; C1q, complement component 1q; SARM1, sterile alpha and Toll/interleukin-1 receptor motif-containing 1; NAD, nicotinamide adenine dinucleotide; PD-1, programmed cell death protein 1; ICOSL, inducible T-cell costimulator ligand; DCs, dendritic cells; NETs, neutrophil extracellular traps; SCFA, short-chain fatty acid.

**Table 2:** Evidence supporting metabolic interventions in rheumatoid arthritis.

Intervention	Stage and Evidence	Limitations	Measures Still Needed
HK2/PFKFB3/choline kinase	Preclinical. Patient-derived FLS and experimental arthritis [53,54,57]	Preclinical; protect metabolically deficient T-cell states	Target engagement, invasion and survival
GLS1/GLUD1	Preclinical. FLS GLS1 perturbation; macrophage nutrient adaptation [15,56]	Distinct enzymes and cell functions; no selective clinical validation established here	Substrate use, proliferation and antigen presentation
SLC5A12	Preclinical. RA-relevant T-cell and arthritis experiments [16]	Preclinical; transporter and recipient-cell selectivity	Lactate uptake and migration with pH controls
SUCNR1	Preclinical. Macrophage/DC and arthritis experiments [82,84,85]	Preclinical; recent mitochondrial mechanism lacks established independent replication	Receptor response and joint or lymph-node function
Itaconate/ACOD1	Preclinical. Native metabolite, derivative and genetic studies [78,91,92]	Bone findings differ across studies; derivative specificity	FLS function, bone resorption and bone formation
SARM1 inhibition	Preclinical. Human RA macrophages and experimental synovitis [29]	Recent preclinical evidence; clinical benefit unresolved	NAD preservation and inflammatory cell death
TNF blockade + ferroptosis induction	Preclinical. Combination benefit in CIA models [58]	Preclinical evidence for selective cell depletion	Fibroblast death, disease activity and tissue damage
Metformin adjunct	120-patient double-blind trial over 12 weeks; 66-patient single-blind trial over 6 months. Short-term clinical improvement [131,132]	Exploratory clinical signal; low MTX dose and attrition; synovial metabolic target engagement unproven	Clinical response, tolerability and tissue mechanism
Everolimus + MTX	121-patient double-blind trial; 12-week ACR20 response 36.1% versus 16.7% with placebo [133]	Exploratory clinical signal with toxicity; tissue mechanism and durability unproven	Clinical response, exposure and tolerability
Low-dose sirolimus	62-patient open-label randomized study, 24 weeks [134]	Exploratory clinical signal; Treg counts do not establish tissue suppression or metabolic engagement	Clinical response and tissue Treg function
NIR-controlled HO-1 delivery	Preclinical. Nanoparticle/gene-delivery study in CIA [136]	Experimental delivery; light, carrier and exposure effects	Local expression, cellular function and biodistribution
Multicomponent lifestyle programme	Randomized 16-week RA trial, 83 participants [137]	Diet, activity and stress effects cannot be separated; not proof of a tissue mechanism	Arthritis and cardiometabolic outcomes; component-specific effects
Ketogenic diet	RA evidence mainly mechanistic or ketosis-adjacent in a 2026 scoping review [138]	RA efficacy and durable reprogramming unestablished	Verified ketosis; active comparator; weight and tissue-mechanism controls

Stages distinguish preclinical target testing, exploratory clinical outcomes and nutritional evidence. Proposed measures are not validated response biomarkers. RA, rheumatoid arthritis; HK2, hexokinase 2; PFKFB3, 6-phosphofructo-2-kinase/fructose-2,6-bisphosphatase 3; GLS1, glutaminase 1; GLUD1, glutamate dehydrogenase 1; SLC5A12, solute carrier family 5 member 12; SUCNR1, succinate receptor 1; ACOD1, aconitate decarboxylase 1; SARM1, sterile alpha and Toll/interleukin-1 receptor motif-containing 1; TNF, tumour necrosis factor; MTX, methotrexate; CIA, collagen-induced arthritis; NIR, near-infrared; HO-1, haem oxygenase 1; FLS, fibroblast-like synoviocytes; DCs, dendritic cells; NAD, nicotinamide adenine dinucleotide; Treg, regulatory T cell; ACR20, at least 20% improvement according to the American College of Rheumatology criteria.

## Data Availability

No new data were created or analyzed in this study. Data sharing is not applicable to this article.
